# Traveling with Terri: bacterial communities

**DOI:** 10.1101/gad.350469.123

**Published:** 2023-01-01

**Authors:** Susan Gottesman

**Affiliations:** Laboratory of Molecular Biology, National Cancer Institute, Bethesda, Maryland 20892, USA

One of the hidden pleasures of life as a scientist is the connections to colleagues who become friends and who remain both colleagues and friends over decades. Terri Grodzicker is one of those people who seem to do this effortlessly and enthusiastically, serving as the hub of an amazing group of scientists whom she has met and befriended, and collecting those who have helped keep *Genes & Development* in top form for the past many years.

## Social science networks: alumni of the Beckwith lab

I met Terri when we overlapped (1969–1971) as early members of the Beckwith lab at Harvard Medical School. I was a graduate student, working on the arabinose operon and isolating tools (transducing phage, before the advent of recombinant DNA) to study how it was regulated (Gottesman and Beckwith 1969). Terri was a postdoctoral fellow, having completed her PhD at Columbia University with David Zipser, working on polarity in the *lac* operon. In Jon's lab, Terri published two papers that provided the foundations for our understanding of how cyclic AMP and catabolite effector protein (“CAP” in the Beckwith lab; “CRP” at NIH and elsewhere) interacted with the *lac* regulatory region (Beckwith et al. 1972; Arditti et al. 1973). I left for a postdoctoral fellowship at NIH in 1971, adding bacteriophage λ to my bacterial genetics repertoire; bacterial regulatory circuits are still the focus of my research. Terri was more adventurous, heading for Cold Spring Harbor Laboratory and a switch from bacteria to adenovirus.

It was easy to keep in touch early on, when the yearly “Phage Meetings” kept me visiting CSHL most summers and we had an opportunity to catch up at wine and cheese receptions or at meals in Blackford. Terri traveled too—and visited us in Bethesda on more than one occasion (including a memorable one where she needed to hide in the downstairs bedroom for time for her posthypnosis follow-up to quit smoking).

Our most memorable chances to hang out together were the every-3-year reunions of the growing Beckwith lab members and alumni, as well as the Beckwith scientific grandchildren working in alumni labs. This symposium involved a few days of talks that certainly captured exciting science, heavily but not exclusively bacterial, tracking how the science of members of the Beckwith lab had evolved. This group and guests (Jon's collaborators and pals) covered a lot of the exciting research in bacterial genetics over the last decades. Although I don't know how many of these stories ended up as *G&D* publications, I can find multiple important papers there from core members of this community. Certainly many of my own major manuscripts ended up here (see below). The image from a T-shirt from the 2017 meeting summarizes those who spent time in the Beckwith lab (*see the image at the top of the next page*).

**Figure:**
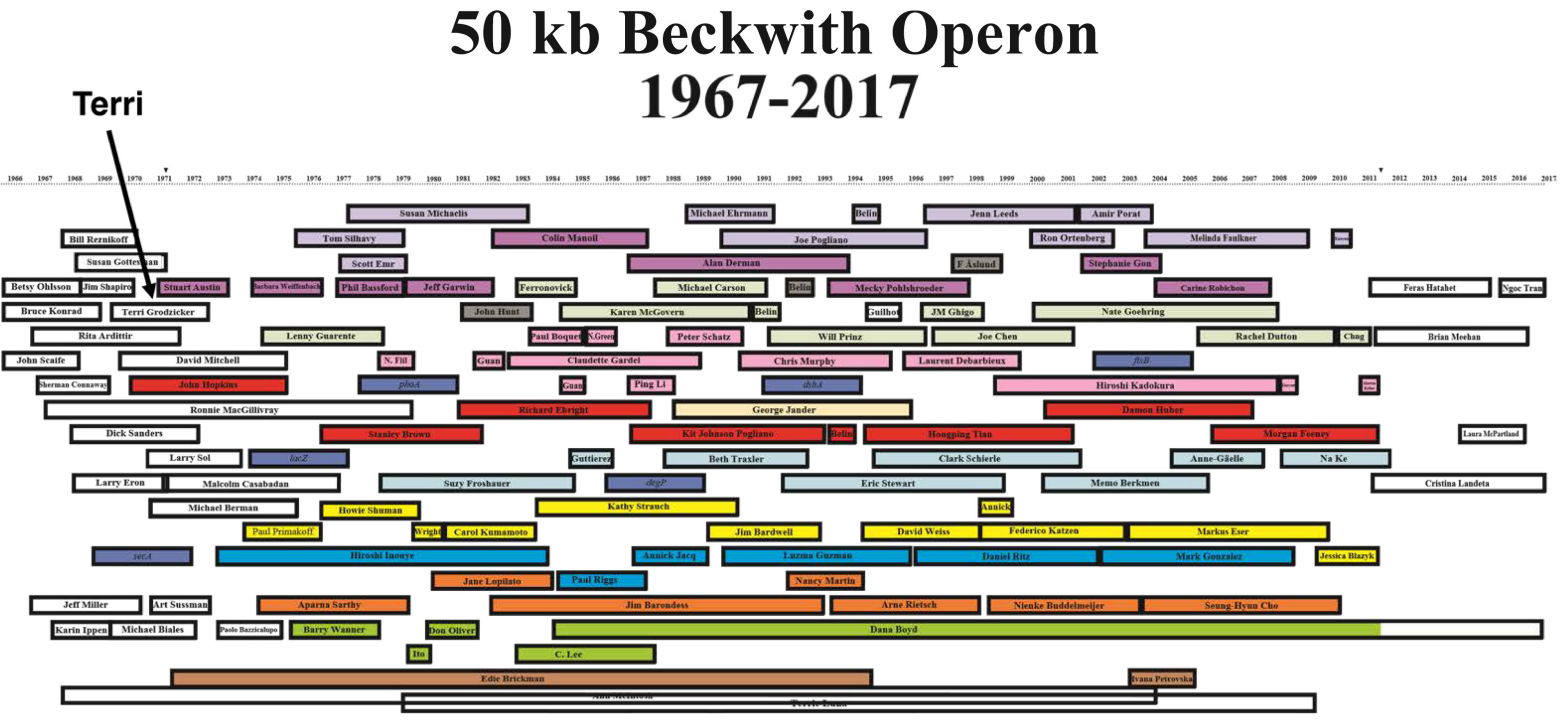
*The image from the T-shirt from the 2017 meeting summarizing those who spent time in the Beckwith lab*.

Starting from “Bacterial Secretion” meetings held at Woods Hole, the Beckwith reunion conferences first widened to include those of us who had worked in the lab presecretion studies and then, in 1999, left Woods Hole, never to return. The 1999 meeting took place in what had been a monastery on the edge of a cliff in Amalfi, a site Jon Beckwith had visited years before and imagined having a conference in. Future meetings were in Europe or in far-flung sites in the U.S. For me, planning for these meetings frequently started with a query from Terri—was I going? Frequently the answer for both of us was yes. That meant a chance for us to explore parts of the world we might not otherwise have gotten to, or to revisit places (Boston) where we had first met, and see old friends and colleagues, all while catching up on exciting science (*see the photos at the bottom of the next page*).

**Figure:**
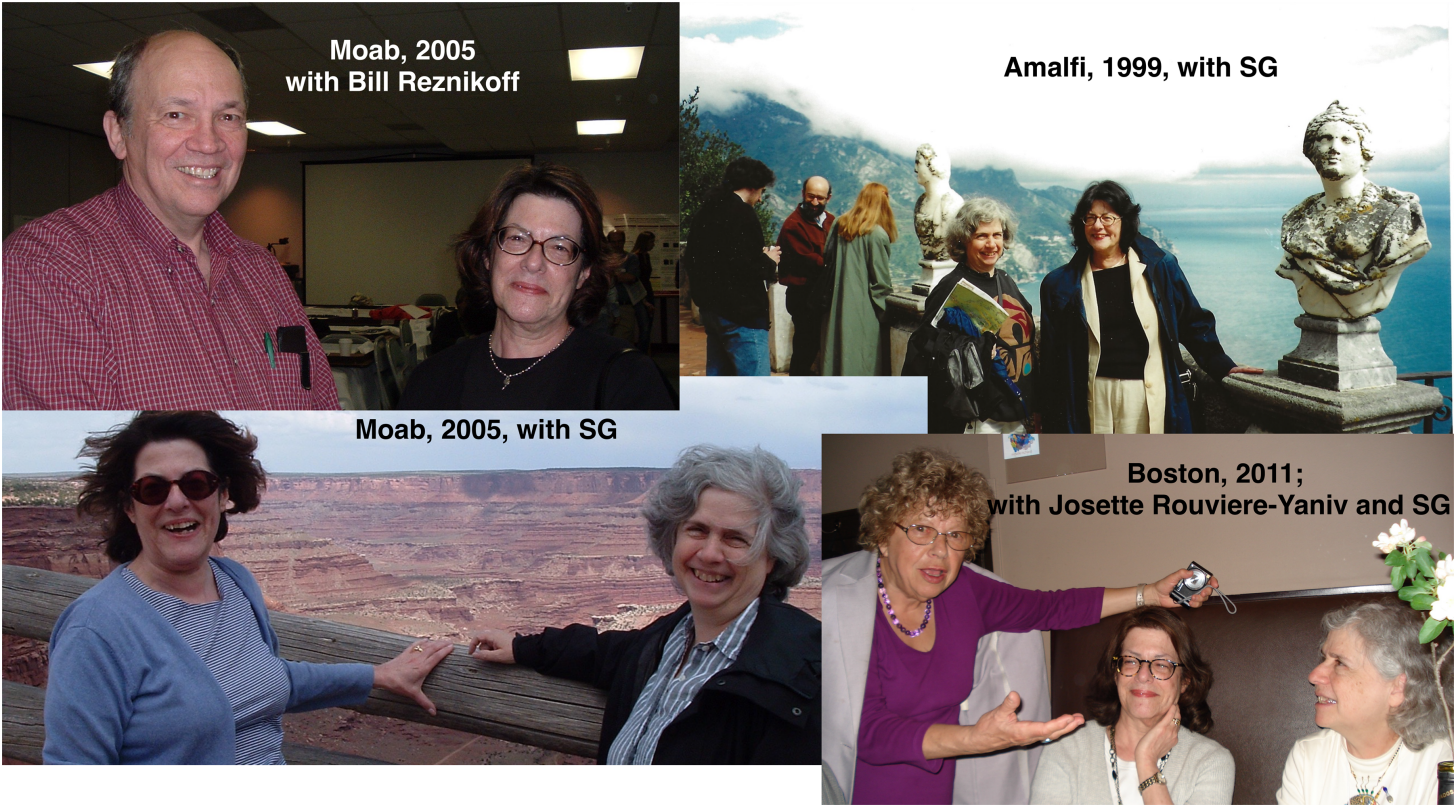
*Old friends and colleagues catching up over the years*.

## Bacterial research and *G&D*

I was invited to join the Editorial Board of *G&D* in 1992, and it has remained as my longest-lasting editorial association. There are many reasons I keep doing this while I manage to get rid of jobs at other journals after a couple of terms. Most importantly, the papers I receive to review are essentially always worth the time it takes to read and review them, are appropriate to my expertise, and are fun to read. Even those I am asked to weigh in on (are they worth a full review?) are generally at a higher level than the majority of papers I get from other journals, including those with single names. I imagine this partially reflects educating the authors over the years about what is worth submitting, but undoubtedly this mostly reflects having a smart, broadly knowledgeable Editor at the helm doing the initial triage as well as evaluating the reviews. For those of us doing work on basic mechanisms in bacterial genetics, cell biology, and development, explaining to Editors of nonbacterial journals why what we do is important for their broad audience can be a real challenge. *G&D*, under Terri's direction, has been a place to publish significant microbial research work in its appropriate full form. The fact that *G&D* has managed to play a similarly important role in many other fields is due to Terri's clear-eyed decisions through the years.

Of course, I've taken advantage of *G&D* in publishing my own work. Looking back as I prepared for this essay, I realize that many of my “best sellers” were in *G&D*.

I started my independent career trying to understand energy-dependent protein degradation in bacteria and how it served in a regulatory role. Our first major review on that was published in *G&D* in 1997 (Gottesman et al. 1997). I visited Bob Sauer at MIT just as they were getting ready to publish the discovery of a novel RNA that acted as both a tRNA and a mRNA (tmRNA), allowing it to direct the cotranslational tagging of truncated polypeptides, directing them to proteolysis (Keiler et al. 1996). The resulting collaboration to identify the proteases responsible for this degradation was published in *G&D* (Gottesman et al. 1998). This is a favorite of mine, since I got to do a lot of the experiments myself, and it combined the work we had done defining proteolysis pathways in bacteria with a new direction our lab was starting to explore—identification and roles of regulatory RNAs in bacteria. Our first global search for small RNAs in *E. coli*, in collaboration with Gisela Storz's lab at NIH, appeared in *G&D* in 2001 (Wassarman et al. 2001). There were other such searches published that year, at least one of which I was asked to review for another journal. I remember trying to explain to the Editor why this was important to publish, even if we didn't yet understand what these small RNAs did. Terri seemed to understand that without us having to spell it out. These papers ended up founding a new field. Again, the first major review I published on this was in *G&D* (Gottesman 2002). The papers we've published in *G&D* in the past 20 years have continued to be ones that I am particularly proud of. Their characteristics: complex stories that require space to explain fully and may not have a very simple take-home message, but that we return to again and again.

## Journal Editors like Terri are critical to the scientific enterprise

A journal that looks at the quality of the work, provides in-depth and careful reviews even when they are a bit painful to deal with, and keeps the science at the forefront of decisions about publishing is an incredibly important part of the scientific enterprise. Implicit in this is the willingness of the Editor to choose knowledgeable reviewers and then to place the comments of the reviewers in context, making tough decisions on the basis of the science rather than what might be currently fashionable. More and more journals seem to be bypassing this critical editorial step—in some cases, just taking all reviews at face value or proposing to leave the readers to decide whether a paper is worthwhile/interpret reviews on their own. Maybe those readers with as much depth of understanding as Terri has can do this effectively, but the quality and longevity of the science that has been published in *G&D* over the past 35 years very much suggest that this is a successful model that is not in need of major change (except for trying to clone Terri to carry the journal through future decades).
